# Effect of flavonoids from grape seed and cranberry extracts on the microbiological activity of *Streptococcus mutans*: a systematic review of in vitro studies

**DOI:** 10.1186/s12903-024-04263-0

**Published:** 2024-06-05

**Authors:** Jeison Stiven Castellanos, Diego Enrique Betancourt, David Díaz-Báez, Paula Alejandra Baldión

**Affiliations:** 1 Departamento de Salud Oral, Facultad de Odontología, Universidad Nacional de Colombia. Av, Cra 30 No. 45-03, Edificio 210, Of. 311, Bogotá, Colombia; 2 Unit of Oral Basic Investigation – UIBO, School of Dentistry, Universidad El Bosque, Bogotá, Colombia

**Keywords:** *Streptococcus mutans*, Grape seed extract, Dental caries, Cranberry, Proanthocyanidin

## Abstract

**Objective:**

To provide an overview of the available scientific evidence from in vitro studies regarding the effect induced by the flavonoids contained in grape seed extracts (GSE) and cranberry on the microbiological activity of *Streptococcus mutans* (*S. mutans*).

**Methods:**

This systematic review was performed following the parameters of the PRISMA statement (Preferred Reporting Items for Systematic Reviews and Meta-Analysis). Electronic and manual searches were conducted using PubMed, ScienceDirect, Web of Science, EBSCO, and Cochrane databases. Reference lists of selected articles were reviewed to identify relevant studies. The search was not limited by year and was conducted solely in English. Eligible studies comprised publications describing in vitro studies that evaluated the effect of flavonoids derived from GSE and cranberry extracts on the microbiological activity of *S. mutans*. Common variables were identified to consolidate the data. Authors of this review independently screened search results, extracted data, and assessed the risk of bias.

**Results:**

Of the 420 studies identified from the different databases, 22 publications were finally selected for review. The risk of bias was low in 13 articles and moderate in 9. The studies analyzed in this review revealed that cranberry extract has an inhibitory effect on the bacterial growth of *S. mutans* in ranges from 0.5 mg/mL to 25 mg/mL, and GSE exerts a similar effect from 0.5 mg/mL to 250 mg/mL. Additionally, the extracts or their fractions showed reduced biofilm formation capacity, decreased polymicrobial biofilm biomass, deregulation of glycosyltransferases (Gtf) B and C expression, and buffering of pH drop. In addition to adequate antioxidant activity related to polyphenol content.

**Conclusions:**

The overall results showed that the extracts of cranberry and grape seed were effective in reducing the virulence factors of the oral pathogen. According to the data, proanthocyanidins are the active components in cranberry and grape seed that effectively resist *S. mutans*. They can inhibit the formation of insoluble polysaccharides in the extracellular matrix and prevent glycan-mediated adhesion, cohesion, and aggregation of the proteins in *S. mutans*. This suggests that these natural extracts could play an important role in the prevention of cariogenic bacterial colonization, as well as induce a decrease in their microbiological activity.

**Supplementary Information:**

The online version contains supplementary material available at 10.1186/s12903-024-04263-0.

## Introduction

Dental caries is a biofilm-mediated disease that has been described as a highly dynamic, multimicrobial, diet-induced disease process [1]. Carious biofilms develop because pathogens accumulate on tooth surfaces, forming highly structured microbial communities that adhere tightly and organize in an extracellular matrix [2]. *Streptococcus mutans* (*S. mutans*) is a Gram-positive, facultative anaerobic, catalase-negative bacterium that produces lactic acid and can reduce the pH of the environment from 7 to 4.2 in approximately 24 h. Additionally, it is capable of fermenting and producing acids from carbohydrates such as glucose, lactose, and raffinose, among others, which is why it is implicated as the key pathogen in the development of dental caries. *S. mutans* is capable not only of carbohydrate breakdown, but also of forming glucans that are important for interacting with tooth structure [3]. By metabolizing carbohydrates such as sucrose, intracellular (IPS), and extracellular (EPS) polysaccharides are synthesized. On the one hand, IPS provides bacteria with an endogenous nutrient source of carbohydrates, during periods when they are deprived of nutrients, to continue acid formation. On the other hand, the synthesis of EPS promotes the adhesion and accumulation of bacteria on the tooth surface and causes structural changes such as increased porosity in dental biofilms [2, 3].

The EPS matrix creates cohesive biofilms that adhere to surfaces, while protecting enclosed pathogens from antimicrobials, making them difficult to treat or remove [3, 4]. The virulence of *S. mutans* is related to the expression of Glucosyltransferases (Gtf), enzymes that are responsible for the synthesis of extracellular glucan polymers from sucrose. These enzymes cover the dental structure with glucans to promote adhesion, by means of surface proteins that contain glucan-binding properties and allow the formation of an extracellular matrix that has the function of anchoring the biofilm [5]. In order to bind with the dental structure, the surface adhesins of *S. mutans*, called antigens I and II, interact with salivary proteins to form a biofilm, which will be anchored to the dental structure [6]. Once the dental biofilm composed of *S. mutans* is formed, due to a prolonged intake of carbohydrates and the porosity caused by the EPS, the sugars penetrate the deepest parts of the biofilms. This allows the microorganism to induce an acidic extracellular environment that helps to reduce its vulnerability, given its virulence factors. This process is the basis of its cariogenic action since it gives rise to an imbalance that initiates the demineralization process, with a reduction in the concentrations of calcium (Ca), inorganic phosphorus (Pi) and fluorine (F), associated with the dissolution of mineral deposits or the inhibition of their storage [3].

Inadequate oral health is particularly predictive of multiple pathological outcomes, including dental caries [1, 7]. Caries is a highly predictable and avoidable condition; however, it remains the most prevalent chronic disease in children and adults worldwide [8, 9]. It is estimated that three billion people have dental caries in the world, and that between 60 and 100% of children and adults suffer from caries at some point in their lives [10]. For this reason, it is necessary to develop and refine different strategies to prevent and solve it. The most used prevention method is the application of different forms of fluoride; however, exploring new alternatives to prevent, stop and restore caries with the help of natural agents can help to reduce caries rates in the population. Biofilm control approaches that disrupt the EPS production and thereby compromise the ability of *S. mutans* to assemble and maintain biofilms on tooth surfaces could be potentially effective alternatives to antimicrobials [11].

Grape seed extract (GSE) and cranberry (*Vaccinium macrocarpon*) are readily available plant-based supplements. Their extracts are rich in polyphenols comprising flavonoids such as proanthocyanidins (PACs) and they have promising biological properties, such as antioxidant and antimicrobial action. The association between PACs and oral health has been demonstrated in the literature through their antimicrobial and antiadherent action against the pathogen *S. mutans*, which leads to reduction in the formation of insoluble polysaccharides in the matrix.

For this reason, the objective of this review is to collect and analyze the available evidence regarding the effect of the flavonoids contained in grape seed and cranberry extracts on the microbiological activity of *S. mutans*, for the prevention of dental caries.

## Methods

This systematic review was performed following the parameters of the PRISMA statement (Preferred Reporting Items for Systematic Reviews and Meta-Analysis) [12]. Based on the study population, intervention, comparison, and outcome, the research question was posed as follows: What is the effect generated by the extracts of grape seeds and cranberry on the microbiological activity of *S. mutans*?

### Eligibility criteria

The criteria for inclusion in this study were as follows: (1) publications of in vitro studies that evaluated the effect of flavonoids from grape seed and cranberry extracts on the microbiological activity of *S. mutans*; (2) in vitro studies that evaluated the growth, coaggregation, and formation of biofilms with *S. mutans* by exposure to flavonoids contained in GSE and cranberry extracts; (3) in vitro studies comparing the effect of flavonoids from GSE and cranberry extracts with the effect of other antimicrobial agents on the activity of *S. mutans*.

The criteria for exclusion from the study were as follows: (1) literature review studies, systematic reviews, meta-analyses, and case reports; (2) studies that reported the crosslinking effect of flavonoids on dentine collagen or their ability to inhibit matrix metalloproteinases to stabilize the hybrid layer; (3) publications that did not report the number of replicates per experiment or that reported a number of replicates less than 3 per test group; (4) studies that reported the remineralizing effect of flavonoids without reporting their effect on the microbiological activity of *S. mutans*.

### Outcomes of interest

The outcomes of interest in the present study were: (1) bacterial proliferation of *S. mutans* in samples exposed to GSE and cranberry extract; (2) the impact of exposure to GSE and cranberry on biofilm formation with *S. mutans*; (3) concentrations and effective times of GSE and cranberry for the inhibition of the microbiological activity of *S. mutans*.

### Data sources and search strategy

An electronic search was performed in the following databases: PubMed, Elsevier, Scopus, ScienceDirect, Web of Science, EBSCO, and Cochrane. For searching the articles, there was no year restriction, and the search was limited to articles written in the English language. The latest search was performed to include studies published up to December 30, 2023. Keywords and their combinations used in the searches included: *Streptococcus mutans*, biofilm, dental caries, proanthocyanidins, grape seed extract, lingonberry extract, and cranberry extract (Table 1 and Additional file 1). In addition, to determine the relevance of other articles that met the inclusion criteria, the references of all included articles were also searched. To find unpublished data, we also searched the database listing unpublished studies (OpenGray).

**Table 1:** Search algorithms in the different databases

Databases	Search algorithm	Limitation
PubMed	(((((Streptococcus mutans[MeSH Terms]) OR (Streptococcus mutans)) OR (Biofilms[MeSH Terms])) OR (Biofilm*)) AND (((((((Grape Seed Extract[MeSH Terms]) OR (Grape Seed Extract)) OR (Grape Seed*)) OR (cranberry extract*)) OR (lingonberry extract*)) OR (lingonberry)) OR (cranberry))) AND (((((((((microbiological activity) OR (Minimum Bactericidal Concentration)) OR (microbial sensitivity tests[MeSH Terms])) OR (Minimum Inhibitory Concentration)) OR (Microbial Sensitivity Tests)) OR (Concentration, Minimum Inhibitory)) OR (Dental caries[MeSH Terms])) OR (dental caries)) OR (Dental Decay))	Language: English
Scopus	( ( TITLE-ABS-KEY ( ( "streptococcus mutans" OR "s. mutans" OR "biofilm" OR "biofilms" OR "dental plaque" OR "dental biofilms" OR "dental caries"))) AND ( TITLE-ABS-KEY ( ( "cranberry" OR "lingonberry" OR "lingonberry extract*" OR "cranberry extract*" OR "grape seed*" OR "grape seed extract")))) AND ( TITLE-ABS-KEY ( "microbiological activity" OR "minimum bactericidal concentration" OR "microbial sensitivity tests" OR "minimum inhibitory concentration" OR "microbial sensitivity tests" OR "concentration minimum inhibitory"))	
Science Direct	("streptococcus mutans" OR Biofilm OR "dental caries") AND (proanthocyanidins OR "grape seed extract" OR "lingonberry extract" OR "cranberry extract")	Research Article Thematic area: medicine and dentistry. Immunology and Microbiology
Web of Science	(AB = (("streptococcus mutans" OR Biofilm OR "dental caries") AND (proanthocyanidins OR "grape seed extract" OR "lingonberry extract" OR "cranberry extract")))	Advanced search Language: English
EBSCO	("streptococcus mutans" OR Biofilm OR "dental caries") AND (proanthocyanidins OR "grape seed extract" OR "lingonberry extract" OR "cranberry extract")	Source: Dentistry and Oral Sciences
Cochrane	("streptococcus mutans" OR Biofilm OR "dental caries") AND (proanthocyanidins OR "grape seed extract" OR "lingonberry extract" OR "cranberry extract")	
Elsevier	[See Additional file 1*]	

^*^Additional file 1 shows in more detail the search algorithms in each database

### Selection and data extraction

Potential primary studies were identified by examining the titles and abstracts of the investigations. The manuscripts that met the eligibility criteria were fully read. Two authors (JC, PB) reviewed the titles and abstracts to assess compliance with the inclusion criteria. Subsequently, only the abstracts were independently reviewed by two authors to reach a consensus on compliance with the inclusion criteria (JC, PB). In those summaries which led to doubts between the authors, a third evaluator (DB) conducted an additional evaluation. Full-text articles were obtained and reviewed by the two authors (JC, PB), and the final inclusion was made in consensus with the other researcher (DB). Any disagreement was discussed and resolved among the authors (JC, PB, DB). The reference lists of the selected articles were reviewed, and the full texts of the included studies were examined. A protocol for data extraction (PRISMA) was implemented, and data related to the research question were extracted and recorded in duplicate using forms designed for this purpose, taking into account: (1) location, year, and citation of the study; (2) type of study; (3) characteristics of cultures or biofilms; (4) types of interventions or exposure; (5) results obtained; (6) conclusions.

### Assessment of the risk of bias and quality of included studies

The methodological quality of the studies was determined using a tool to assess the risk of bias, adapted by de Almeida et al. [13] based on the methodology used by Cericato et al. [14] with some modifications. The parameters considered are described in Table 2. A score was assigned to each study, classifying them according to their quality as: low quality (0–9 points), moderate quality (10–14 points), or high quality (15–18 points).

**Table 2:** Risk of bias evaluation criteria and the quality of the selected studies

Q	Evaluation criteria	Score
1	Objective, methodology, results and conclusion of the study are clearly articulated	2 points
2	The study sets out a clear and precise objective	2 points
3	Ethical aspects of the research are cited in the text *	2 points
4	Methodology is reported clearly and in detail**	2 points
5	Appropriate control groups were used to make comparisons	2 points
6	Statistical tests and p values are described	2 points
7	The study presents the results clearly and precisely	2 points
8	The limitations of the study are discussed	2 points
9	The conclusions are consistent with the objective of the study	2 points

^*^ The study complies with the approval of the ethics committee, a declaration of project financing and conflict of interest

^**^ Assays used, the number of intra and interexperiment replicates, instruments used, reported brands, references, and concentrations

### Data analysis

The data extracted from the full-text articles are recorded in Tables 3, 4, 5, 6, 7, 8, 9 and 10, which includes the methodology of the included studies. Identification of the common variables in the different articles was made to facilitate the consolidation of the data. A descriptive summary of the variables considered (bacterial inhibition, biofilm formation, antioxidant capacity, expression of GtfB and C, and F-ATPase, morphological changes, pH changes, adhesion, induction of cell death and cytotoxicity) was compiled.

**Table 3:** Characteristics and methodology of 22 articles included in the review

Author	Bacterial strain	Intervention				
Reference	Additional strains	Extract used	Objective to evaluate	Technique type	Substance	Protocol (incubation time and temperature; control and treatment groups)
Kokubu, E.et al. (2019) [24]	*S. mutans* MT8148R Two more bacterial strains	Cranberry and lingonberry	Biofilm formation Bactericidal activity	Crystal violet stain. Spectrophotometric absorbance ATP bioluminescence assay (BacTiterGlo)	Todd Hewitt broth	T = 24 h / T° = 37 °C Control group: No extract Experimental group: G1: 1,000 ml of 5% ethanol G2: 1,000 ml of 70% ethanol
Greene, C. et al. (2020) [25]	*S. mutans*	Cranberry + C_18_H_36_O_2_ + PVP o LAE	Adhesion to sHA* Biofilm inhibition	Olympus SZX10 dissection microscope Crystal violet stain. reader for absorbance	sHA* BHI**	T = 24 h / T° = 37 °C Control group (-): PBS Experimental group: G1: Cranberry G2: Cranberry + C_18_H_36_O_2_ + PVP Proportions: 10:1:1, 10:1:0,5, 10:1:0,25, 10:1:0,125, 10:1:0,0625 y 10:1:0,03125 G2: Cranberry + C_18_H_36_O_2_ + LAE
Swadas, M et al. (2016) [26]	*S. mutans* ATCC 25175	Grape seed	Antimicrobial activity	Colony forming unit count (CFU)	*Mitis salivarius* bacitracin agar	T = 48 h / T° = 37 °C Control group ( +): Chlorhexidine gluconate Control group (-): Ultrapure water Experimental group: G1: 500 mg/mL extract G2: 250 mg/ extract G3: 125 mg/ml extract

*C*_*18*_*H*_*36*_*O*_*2*_ Stearic acid, *PVP *Polyvinylpyrrolidone, *LAE *Ethyl lauroyl arginate

^*^Saliva coated hydroxyapatite

**Brain Heart Infusion

**Table 4:** Characteristics and methodology of 22 articles included in the review

Author	Bacterial Stain	Intervention				
Reference	Additional strains	Extract used	Objective to evaluate	Technique type	Substance	Protocol (incubation time and temperature; control and treatment groups)
Singhal, R. et al. (2022 [27])	*S.* *mutans* MTCC 25175 One more bacterial strain	Cranberry	*MIC * *MBC * Time Kill Assay Biofilm Inhibition Biofilm Morphology	Serial dilution and microcentrifugation Aerobic incubation Colony forming unit count UFC/ml Crystal violet stain Glutaraldehyde fixation, dehydration, scanning electron microscope	BHI** BHI** + sucrose	T = 24 h / 35 °C Control group: Sucrose broth without extract. *MIC MBC:* 0.5 McFarland standard inoculums of respective microorganisms Experimental group: Different concentrations of the extract. *MIC:* 12.5 mg/dL *MBC:* 25 mg/dL against *S. mutans*
Koo, H.et al. (2010) [28]	*S.* *mutans* UA159	Cranberry	Gtfs activity Decreased acidogenicity	Culture and purification with chromatography on hydroxyapatite GtfB activity determined by enzymes in solution or adsorbed on hydroxyapatite beads pH drop across glass electrode	In vitro*:* Discs of sHA* *Biofilm preparation*: Yeast extract and tryptone + sucrose *Mitis salivarius* bacitracin agar	T = 24 h / T° = 37 °C Control group ( +): 250 ppm sodium fluoride Control group (-): 10% ethanol Experimental group: Twice daily topical application of PAC * * * (1,5 mg/mL in ethanol to 10%, v/v)

*MIC *Minimum inhibitory concentration, *MBC *Minimum bactericidal concentration, *Gtfs *Glycosyltransferases

*Saliva coated hydroxyapatite

**Brain Heart Infusion

***Proanthocyanidin

**Table 5:** Characteristics and methodology of 22 articles included in the review

Author	Bacterial Strain		Intervention			
Reference	Additional strains	Extract Used	Objective to evaluate	Technique Type	Substance	Protocol (incubation time and temperature; control and treatment groups)
Sumathi, S et al. (2019) [29]	*S. mutans* Three more bacterial strains	Cranberry	*MIC* Time Kill Assay Antibiofilm activity Bactericidal Activity	Broth microdilution method Modified microdilution and reading with spectrophotometer MTT Assay	*MIC* Mueller–Hinton on agar Antibiofilm activity: Trypticase Soy Broth	T = 24 h / T°37 °C Control group ( +): Ciprofloxacin Control group (-): Distilled water Experimental group: different concentrations of the extract between 1,0 y 25 mg/ml
Philip, N et al. (2019) [7]	*S. mutans* Three more bacterial strains	Cranberry	Decreased acidogenicity EPS/ Microbial biovolumes and organization Microbial counts Biomass biofilm	Lactate dehydrogenase assay Confocal microscopy 3D images Serial dilution Crystal violet assay	sHA* Polymicrobial biofilm: Saliva and glycerol in McBain's medium rich in mucin + 1% sucrose	T = 48 h / T° = 37 °C Control group: PBS Experimental group: 2 ml/well of the extract solution to 500 μg/mL
Abu-obaid, E et al. (2020) [30]	*S. mutans* ATCC 25175 Two more bacterial strains	Cranberry	*MIC* *MBC*	Agar dilution *MIC* range on extended plate	*Mutans-Sanguis* Agar	T = 48 h / 37 °C Control group ( +): Chlorhexidine digluconate with alcohol Control group (-): Distilled water Experimental group: Rinses G1: herbal mix G2: Cranberry G3: Chlorhexidine digluconate

*MIC *Minimum inhibitory concentration, *MBC *Minimum bactericidal concentration

*Saliva coated hydroxyapatite

**Table 6:** Characteristics and methodology of 22 articles included in the review

Author	Bacterial Strain	Intervention				
Reference	Additional strains	Extract Used		Technique type	Protocol (incubation time and temperature; control and treatment groups)	Protocol (incubation time and temperature; control and treatment groups)
Daglia, M. et al. (2010) [31]	*S. mutans* 9102 and S. *mutans* ATCC 25175	Grape seed	*MIC * Adhesion Biofilm formation	Lower concentration that inhibits visible bacterial growth Cell labeling, number of bacteria adhered to HA^«^ Crystal violet staining, absorbance at 540 nm by spectrophotometry	BHIB y BHIA + 0.1 M sucrose	T = 48 h / T° = 37 °C
Duarte, S. et al. (2022) [32]	*S.* *mutans* UA159 One more bacterial strain	Cranberry	Gtfs Activity pH drop	Sucrose glucose incorporation and adhesion to sHA* Labeling of pH decrease with a glass electrode for 2 h	sHA*	Control group ( +): Medium inoculated without DRW
Zhao, W.et al. (2014) [33]	*S. mutans *UA159	Grape seed 97,8% of proanthocyanidin	*MIC* Biofilm *MIC* Polarized light microscopy	Plate microdilution Lower concentration of GSE that inhibits biofilm formation Quatification of the depth of the lesions	sHA*	Control group (-): Uninoculated medium

*MIC *Minimum inhibitory concentration, *Gtfs* Glycosyltransferases, *BHIB *brain heart infusion broth, *BHIA *Brain heart infusion agar, *GSE *Grape seed extract, *DRW *Dealcoholized wine

*Saliva coated hydroxyapatite

**Table 7:** Characteristics and methodology of 22 articles included in the review

Author	Bacterial strain	Intervention				
Reference	Additional strains	Extract used	Objective to evaluate	Technique type	Substance	Protocol (incubation time and temperature; control and treatment groups)
Furiga, A. et al. (2013) [34]	*S. mutans* ATCC 25175 Five more bacterial strains	Grape seed	Inhibitory effect on biofilms Effect on glycosyltransferases *MIC y MBC* Equivalent Antioxidant Capacity of Trolox Cell viability	Saliva centrifugation, aerobic or anaerobic incubation Increased sugar concentration Broth microdilution Slopes of the dose–response curves of the test compound and Trolox Calcein (LIVE/DEAD)	Tryptic soy broth sHA* disks *Mitis Salivarius* Agar + Tellurite	T = 24 h / T° = 37 °C Control group: ultrapure water Experimental group: GSE + Amine Fluoride (Fluorinol-)
Prince, A. et al. (2022) [35]	*S. mutans* ATCC 25175	Cranberry	Synergy	*Checkerboard assay* to verify synergy in a clean laminar flow hood	BHI^**^	T = 18 h—24 h / T° = 37 °C Control group ( +): Chlorhexidine gluconate 2% Control group (-): Distilled water Experimental group: G1: Subcritical Pressed Water Extract (SWP) G2: Subcritical Water Fruit Extract (SWF) G3: Subcritical water extract of pressed cake with tannase (SWPE) G4: resin extract (R) G5: resin extract with tannase (RE)

*MIC *Minimum inhibitory concentration, *MBC *Minimum bactericidal concentration, *GSE* Grape seed extract

*Saliva coated hydroxyapatite

**Brain heart infusion

**Table 8:** Characteristics and methodology of 22 articles included in the review

Author	Bacterial strain	Intervention				
Reference	Additional strains	Extract used	Objective to evaluate	Technique type	Substance	Protocol (incubation time and temperature; control and treatment groups)
Ikai, H. et al. (2013) [36]	*S.* *mutans* JCM 5705	Cranberry	Bactericidal assay	Laser irradiation for 3 min	BHI^**^	T = 48 h / T° = 37 °C Control group: Hydrogen peroxide Experimental group: Extract concentrations from 0 to 8 mg/ml
Feng, G. et al. (2013) [37]	One more bacterial strain *S.* *mutans* UA159	Cranberry	Biofilm accumulation and architecture Bacterial adhesion	3D Confocal Imaging and Fluorescence Imaging Recuento por centelleo	sHA^*^	T = 24 h / T° = 37 °C Control group: 15% ethanol in 2.5 mM potassium phosphate buffer Experimental group: Topical applications of PAC fraction
Philip, N. et al. (2019) [38]	*S.* *mutans* ATCC 25175	Cranberry	Biofilm metabolic activity Acid production Biovolumes and organization Bacterial counts	Colorimetric assay, bioreduction of tetrazolium salt, 2,3-bis Standard curve colorimetric assay measuring absorbance at 340 nm Spectral rotating disk confocal microscope images CFU number using digital colony counter	sHA^*^ BHI^**^ + 0,2% sucrose	T = 24 h / T° = 37 °C Control group ( +): Chlorhexidine 0.12% Experimental group: Four different concentrations of extracts ranging from 62.5 to 500 μg mL

*CFU *Colony forming unit, *PAC *Proanthocyanidin

*Saliva coated hydroxyapatite

**Brain heart infusion

**Table 9:** Characteristics and methodology of 22 articles included in the review

Author	Bacterial strain	Intervention				
Reference	Additional strains	Extract used	Objective to evaluate	Technique type	Substance	Protocol (incubation time and temperature; control and treatment groups)
Gregoire, S et al. (2007) [39]	*S. mutans* UA159	Cranberry	Effects on Glucosyltransferase pH drop F-ATPase activity	Incorporation of labeled glucose sucrose pH labeling with a glass electrode in 75 min Inorganic phosphate release test in a mixture	Tryptic soy broth *Gtfs Activity:* sHA^*^ *pH drop:* 100 mmol Tris-maleate buffer + 5 mmol ATP, 10 mmol MgCl2, permeabilized cells	T = No report / T° = No report Control group: 15% ethanol and 2–5% DMSO Experimental group: G1: Flavonols-quercetin G2: Proanthocyanidins (PACs) epicatechin A monomer and procyanidin dimer G3: Phenolic acids-caffeic acid
Neto, C et al. (2017) [40]	*S. mutans* UA159 Six more bacterial strains	Cranberry	Biofilm formation *MIC* Oxidant uptake capacity	Staining with crystal violet, microplate reader at 595 nm Lower concentration to reduce biofilm Luminol-dependent chemiluminescence assay	BHI^**^ + 2% Sucrose	T = 24 h / T° = 37 °C Control group ( +): Lactose or procyanidin patterns Control group (-): PBS Experimental group: Cranberry concentrate between 500 y 120 μg·ml
Smullen, J et al. (2006) [41]	*S. mutans* 10,499 y R9 Fourteen more strains	Grape seed	MIC MBC Adhesion Antimicrobial activity Bactericidal activity	Agar dilution susceptibility broth dilution Absorbance labeling at 480 nm	BHI^**^ Mueller Hinton Agar	T = 48 h / T° = 37 °C Control group: No extract Experimental group: grape seed extract with 95% phenolic compounds

*MIC *Minimum inhibitory concentration, *MBC *Minimum bactericidal concentration, *Gtfs *Glucosyltransferases

*Saliva coated hydroxyapatite

**Brain Heart Infusion

**Table 10:** Characteristics and methodology of 22 articles included in the review

Author	Bacterial strain	Intervention				
Reference	Additional strains	Extract used	Objective to evaluate	Technique type	Substance	Protocol (incubation time and temperature; control and treatment groups)
Yamanaka, A. et al. (2004) [42]	*S. mutans* MT8148R Six more bacterial strains	Cranberry	Biofilm formation Adhesion	Crystal violet stain Hydrophobicity	TSB sHA^*^	T = 24-72 h / T° = 37 °C Control group: Buffered KCl without cranberry juice Experimental group: G1: 100 μL cranberry juice at 25% of KCl G2: 500 μL cranberry juice at 25% of KCl
Elgamily, HM. et al. (2023 [43]	*S. mutans* Two more bacterial strain	Cranberry	Inhibitory effect on biofilms	Full scan chromatography Hydrolysis and measurement of MS MS spectra	TSB	T = 48 h / T° = 37 °C Control group: No report Experimental group: G1: Cranberry juice G2: Cranberry functional drink
Nowaczyk, PM et al. (2021) [44]	*S. mutans* 10,499 y R9 One more strain	Grape seed	Biofilm morphology Inhibitory effect on biofilms	Electron microscopy	TSB	T = 48 h / T° = 37 °C Control group ( +): sterile saline solution Control group (-): No treatment Experimental group: probiotic-GSE Jelly candy with 50 μg/2 mL of extract

*GSE* Grape Seed Extract, *TSB* Tryptic soy brothhi, *KCl *Potassium chloride

*Saliva coated hydroxyapatite

## Results

### Search results

**Fig. 1:**
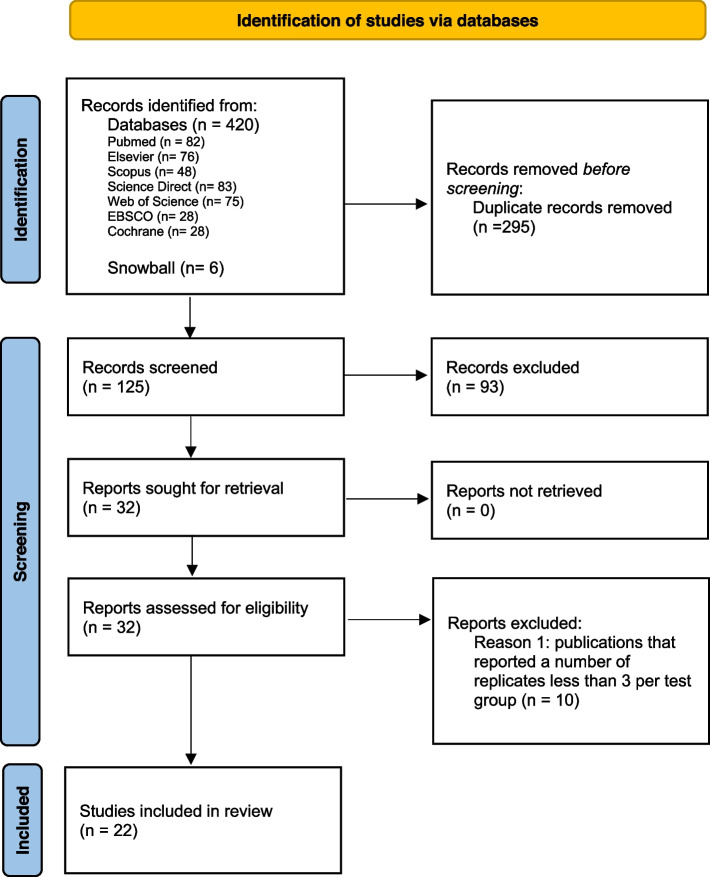
Identification of studies via databases

A total of 420 studies were identified from the different databases, out of which 295 duplicate reports were eliminated; of 125 studies reviewed, 93 studies were excluded due to the title and abstract. Accordingly, 32 publications were selected for full-text reading. Subsequently, ten articles were eliminated for not meeting the criteria of reporting a minimum of three replications per experiment [15–23] and, finally, 22 publications were selected for our review (Fig. 1).

### Risk of bias and quality assessment

The risk of bias of the 22 included studies is presented in Tables 11, 12, 13, 14 and 15. 13 of them presented low risk [59%], 9 studies presented moderate risk (41%), and no study was classified as high risk [0%]. Bias evaluation was performed according to the parameters established in Table 2. The main risk of bias was found in that study limitations were either not reported in the study report [24–26, 29, 36, 37, 39, 41–43] or were described briefly [27, 28, 31–34, 40, 44]. Secondly, there was a failure to include relevant ethical aspects such as institutional ethics committee approval, or patient informed consent for saliva sample collection was not clearly detailed in the article text, or a declaration of project financing and conflict of interest was absent [27–29, 31–37, 39–42, 44]. Thirdly, there was no clear and precise description of the study objective regarding which specific aspects were to be evaluated regarding the effect of the extracts on antimicrobial activity or biofilm formation [7, 24, 26, 27, 35, 37, 38, 40, 42].

**Table 11:** Evaluation of the quality of the articles included

Reference	Q1	Q2	Q3	Q4	Q5	Q6	Q7	Q8	Q9	Total	Risk*
[7] Philip et al. (2019)	2	1	2	2	2	2	2	1	2	16	Low
[24] Kokubu et al. (2019)	2	1	2	2	2	0	2	0	2	13	Moderate
[25] Greene et al. (2020)	2	2	2	2	2	2	2	0	1	15	Low
[26] Swadas et al. (2016)	2	1	2	2	2	1	2	0	1	13	Moderate
[27] Signhal et al. (2020)	2	1	1	2	2	0	2	1	2	13	Moderate
[28] Koo et al. (2010)	2	2	1	2	2	2	2	1	2	16	Low
[29] Sumathi et al. (2019)	2	2	1	2	2	0	2	0	2	13	Moderate
[30] Abu et al. (2020)	2	2	2	2	2	2	2	2	2	18	Low
[31] Daglia et al. (2010)	2	2	1	2	2	1	2	1	2	15	Low
[32] Duarte et al. (2006)	2	2	1	2	2	2	2	1	2	16	Low
[33] Zhao et al. (2014)	2	2	1	2	2	2	2	1	2	16	Low
[34] Furiga et al. (2013)	2	2	1	2	2	2	2	1	2	16	Low
[35] Prince et al. (2022)	1	1	1	2	1	2	2	2	2	14	Moderate
[36] Ikai et al. (2013)	2	2	1	2	2	2	2	0	2	15	Low
[37] Feng et al. (2013)	2	1	1	2	2	2	2	0	2	14	Moderate
[38] Philip et al. (2019)	2	1	2	2	2	2	2	2	2	17	Low
[39] Gregoire et al. (2007)	2	2	1	2	1	2	2	0	2	14	Moderate
[40] Neto et al. (2017)	1	0	1	2	2	1	2	1	2	12	Moderate
[41] Smullen et al. (2007)	2	2	1	2	2	2	2	0	2	15	Low
[42] Yamanaka et al. (2004)	2	1	0	2	1	2	2	0	2	12	Moderate
[43] Elgamily et al. (2023)	2	2	2	2	2	2	2	0	1	15	Low
[44] Nowaczyk et al. (2021)	2	2	1	2	1	2	2	1	1	16	Low

^*^High risk (0–9 points), moderate risk (10–14 points), or low risk (15–18 points)

**Table 12:** Effects induced by cranberry and grape seed extracts on *S. mutans*

		Results							
Reference	Extract used	Bacterial Inhibition	Biofilm formation	Antioxidant Capacity	Effect on Gtfs and F-ATPasa	Biofilm morphological changes	pH drop	Adhesion	Cell death induction
[24]	Cranberry	+ G1: 0,5. 1,0. 2,0 mg/ml + G2: 1,0. 2,0 mg/ml	Reduction + G2: 0,5—1 mg/ml	N/A	N/A	N/A	N/A	N/A	N/A
[25]	Cranberry	N/A	Reduction + G1, G2,G3:0,5—5 mg/ml - G1, G2: 0,05 mg/ml	N/A	N/A	N/A	N/A	There was adhesion to HA disk of the extract in G2 y G3	N/A
[26]	Grape seed	+ + G1: 500 mg/ml, G2: 250 mg/ml - G3: 125 mg/ml	N/A	N/A	N/A	N/A	N/A	N/A	N/A
[27]	Cranberry	*MIC*: 12,5 mg/dL *MBC*: 25 mg/dL	16,67 (± 7.21) mg/dL inhibits 50%—24 h. 20.83 (± 7.21) mg/dL inhibits 70%—24 h	N/A	N/A	> extract: disruption in integrity and structure and agglutination	N/A	N/A	50% reduction after 10 h
[28]	Cranberry	N/A	N/A	N/A	Reduction (35–40%) of extracellular polysaccharides Inhibition (40–70%) of GtfB activity	N/A	Decrease in acidic pH (5.6) compared to control group (5.2)	N/A	N/A
[29]	Cranberry	*MIC*: 60 mg/ml	50 mg/mL inhibits 95,2%—24 h	N/A	N/A	N/A	N/A	N/A	N/A

*MIC *Minimum inhibitory concentration *MBC *Minimum bactericidal concentration, *G1, G1, G3 *experimental groups, *Gtfs *Glucosyltransferases

+ Effect

+  + Greater effect

– minor effect

**Table 13:** Effects induced by cranberry and grape seed extracts on *S. mutans*

		Results							
Reference	Extract used	Bacterial Inhibition	Biofilm formation	Antioxidant Capacity	Effect on Gtfs and F-ATPasa	Biofilm morphological changes	pH drop	Adhesion	Cell death induction
[7]]	Cranberry	N/A	500 μg/ml Inhibits (51%)	N/A	EPS decrease	Reduction (38%) biomass in 96 h	Reduction (44%) lactic acid (6,2 ± 1,9 mM/L)	N/A	N/A
[30]	Cranberry	*MIC*, *MBC*: higher in G4. G2 y G3 Similar	N/A	N/A	N/A	N/A	N/A	N/A	N/A
[31]	Grape seed	*MIC*: 19,07 mg/mL *DRW* 2,53 mg/mL *SPE F4*	0,125 mg/mL inhibits (89 ± 4%)	N/A	N/A	N/A	N/A	Inhibition (81,5 ± 5%) SPE F4 a 0,125 mg/ml (25,0 ± 1%) DRW a 0,25 mg/mL	N/A
[32]	Cranberry	N/A	N/A	N/A	Inhibition G3: (85%) of F-ATPase activity *GtfsB:* G1: (50%) G2: (45%) G3: (70%) G4: (40%)	N/A	Final pH (4,7–4,9). pH of control group (3,7)	N/A	N/A
[33]	Grape seed	+ 4 mg/mL	inhibition + 4 mg/dL	N/A	N/A	N/A	N/A	N/A	N/A
[34]	Grape seed	*MIC*: 1000 μg/ml *MBC*: 4000 μg/ml	inhibition + 2000 μg/ml GSE + 10,2 mg/ml Fluorinol	High antioxidant capacity of GSE (6,99 μg/ml)	Inhibition (43,9%) GSE (65,7%) GSE + Fluorinol	2000 μg/ml) Decrease in microcolonies and thickness of biofilm	N/A	N/A	Low level of cell damage

*MIC *Minimum inhibitory concentration, *MBC *Minimum bactericidal concentration, *G1, G1, G3 *experimental groups, *Gtfs *Glucosyltransferases, *GSE *Grape seed extract, *DRW *dealcoholized wine, *SPE F4 *Polymeric proanthocyanidin/15 mL metanol, *EPS *Exopolysaccharides

+ Effect

+  + greater effect

– Minor effect

**Table 14:** Effects induced by cranberry and grape seed extracts on *S. mutans*

		Results							
Reference	Extract used	Bacterial Inhibition	Biofilm formation	Antioxidant Capacity	Effect on Gtfs and F-ATPasa	Biofilm morphological changes	pH drop	Adhesion	Cell death induction
[35]	Cranberry	*MIC* G3, G5: 0,75 mg/ml G4: 0,5 mg/ml	N/A	N/A	N/A	N/A	N/A	N/A	N/A
[36]	Cranberry	+ 2 mg/ml + 80 y 320 nM H_2_O_2_ photo-irradiated	Inhibition + + (8 mg/mL)	N/A	N/A	N/A	N/A	N/A	N/A
[37]	Cranberry	N/A	Inhibition (4.31 ± 2.68) μm3	N/A	Decrease (19.88 ± 7.23) μm3	Accumulation and faulty architecture	N/A	Inhibition of glucan synthesis	N/A
[38]	Cranberry	N/A	Inhibits (32%) a 500 μg/ml. (29%) a 250 μg/ml. (14%) a 125 μg/ml	N/A	N/A	Biomass decrease	Lactic acid reduction (46%) to 500 μg/ml	N/A	N/A
[39]]	Cranberry	N/A	N/A	N/A	Inhibition F-ATPasa: (18–33%) to 500 μmol GtfsB: (20–35%) to 500 μmol	N/A	pH Drop interruption	N/A	N/A
[40]	Cranberry	N/A	Reduction to (320 µg/ mL) NDM raw (80 µg/mL) NDMac	N/A	N/A	N/A	N/A	N/A	N/A
[41]	Grape seed	*MIC*: 0,5 mg/mL	Biofilm reduction in 30 min	N/A	N/A	N/A	N/A	Inhibited 5 mg/mL	N/A

*MIC *Minimum inhibitory concentration, *G1, G1, G3 *experimental groups, *Gtfs *Glucosyltransferases, *NDM* non-dialyzable cranberry, *NDMac* with acetone

+ Effect

+ + greater effect

**Table 15:** Effects induced by cranberry and grape seed extracts on *S. mutans*

		Results							
Reference	Extract used	Bacterial Inhibition	Biofilm formation	Antioxidant Capacity	Effect on Gtfs and F-ATPasa	Biofilm morphological changes	pH drop	Adhesion	Cell death induction
[42]	Cranberry	N/A	Inhibition at (100 μg/mL and 500 μg/mL)	N/A	N/A	N/A	N/A	Reduction 40–60% primary colonization	N/A
[43]	Cranberry	Inhibition G1: (0,5 mL/mL and 0,25 mL/mLG2:0,50 mL/Ml	N/A	N/A	N/A	N/A	N/A	N/A	N/A
[44]	Grape seed	N/A	Reduction of 60% (50 Μg/2 mL) GSE probiotic candy	N/A	N/A	Altered structure	N/A	N/A	N/A

*G1, G2 *experimental groups, *Gtfs* Glucosyltransferases

+ Effect

### Description of studies

#### Location of the studies

Of the included studies, three were conducted in Japan [24, 36, 42], seven in the United States [25, 28, 32, 33, 35, 37, 39], three in India [26, 27, 29], two in Australia [7, 38] one in Saudi Arabia [30], one in Italy [31], one in France [34], one in Israel [40], one in England [41], one in Poland [43], and one in Egypt [44] sixteen were conducted at universities [7, 24–26, 28, 30–35, 37–41], and six in other types of institutions [27, 29, 36, 42–44].

### Experimental models

#### Specimens and bacterial culture

The reported sample size was determined for each study method. In evaluating the biofilm formation, all studies reported having performed the trials at least in triplicate. All studies evaluated the activity of PAC on *S. mutans* [7, 24–44], and ten studies evaluated its activity on two or more bacterial strains [7, 24, 27, 29, 30, 36, 41–44]. Several studies have used certified American Type Culture Collection (ATCC) bacterial strains of *S. mutans* to assess the antibacterial activity of test substances. These strains meet quality criteria such as: i) they were obtained from a certified collection, ii) they were isolated from cultures of the reference strains, and iii) they are identical strains obtained from a subculture of the reference strains. The most used strain was *S. mutans* ATCC 25175 [26, 31, 34, 35, 38, 44]. The second-most used strain was *S. mutans* UA159 [28, 32, 33, 37, 40], which is a naturally competent strain and contains all essential genes for competition and quorum sensing [45]. Kokubu et al. [24] and Yamanaka et al. [42] used *S. mutans* MT8148R15; Daglia et al. [31] used the strain *S. mutans* 9102 in addition to ATCC 25175, Ikai et al. [36] used strain *S. mutans* JCM 5705, and Smullen et al. [41] used the *S. mutans* 10,499 strain. For their part, Duarte et al. [32] and Gregoire et al. [39] used *S. mutans* WHB 410. Some studies do not specifically report the strain used [29, 43] or biofilm formation was performed by collecting saliva from healthy donors [7, 30].

#### Culture protocols

The evaluation of biofilm formation was conducted using various methods and culture media, including Todd Hewitt broth in 96-well cell culture plates [24]; brain–heart infusion (BHI) broth in 96-well cell culture plates [36]; BHI broth + 1% glucose in 96-well cell culture plates [25]; BHI broth + sucrose in 24-well tissue culture plates [27, 38], or in 96-well cell culture plates [31, 40], or in 6-well tissue culture plates [33]; *Mitis Salivarius* agar + tellurite [34]; tryptone-yeast extract broth [44] by the addition of sucrose on saliva-coated hydroxyapatite disks [28, 32, 37]; trypticase soy broth (TSB) in 96-well titre plates [29, 42], or TSB + 20% glycerol [39]. In one study [43], a biofilm growth medium containing a mixture of pasteurized saliva, amino acids, vitamins, nucleotides, inorganic salts, trace elements, and glucose, supplemented with fluid universal medium, and enriched with sucrose and glucose was used. The antibacterial activity against *S. mutans* was also evaluated by different methods, including inoculation on *Mitis salivarius* bacitracin agar medium in Petri dishes [26], using the disc diffusion method (Kirby–Bauer) on agar plates, and the bacterial strains were grown in *Mutans-Sanguis* agar (Hi media) [30], or the cultures of *S. mutans* were inoculated into Mueller–Hinton broth [41]. Sample storage time ranged between 20 and 48 h; in ten studies the sample was incubated for 24 h [24, 27, 29, 32–35, 38, 40, 42], in nine studies, incubation was done for 48 h [7, 25, 26, 30, 31, 36, 41, 43, 44], and in one for 20 h [37]. The temperature reported in twenty studies was 37 °C [7, 24–26, 28–38, 40–44], in one was 35 °C [27], and another study did not report temperature data [39].

#### Extracts used for microbiological tests

For the evaluation of the antimicrobial effect, the studies used different sources of flavonoids. On the one hand, some studies started with fresh cranberry extracts [27–30, 32, 37, 39, 43], from commercially obtained cranberry juice concentrates [7, 24, 25, 30, 35, 38, 40–42], or from samples of red wine and concentrated or distilled white wine [41] or dealcoholized red wine [31]. In contrast, other studies used GSE from fresh red grapes [26] or extracts of GSE obtained from the seeds of *Vitis vinifera* in commercial presentation [26, 33, 44], or the previously purified PACs in commercial presentations [36].

#### Obtaining and characterizing the extracts and fractions rich in polyphenols

For the separation of the fraction rich in polyphenols, they used chromatography [24, 28] and the total polyphenols in each fraction were determined by the Folin-Ciocalteu Method [24, 40]. Greene et al. [25] microencapsulated concentrated cranberry extract in poly(lactic-co-glycolic) (PLGA) using a double emulsion manufacturing procedure. Swadas et al. [26] and Singhal et al. [27] used the maceration method of the previously dried grape seeds or cranberry fruits, as did Abu-obaid et al. [30]; the preparation was carried out in a hydroalcoholic solvent with a ratio of ethanol (70%): water (30%) [26, 27] and subsequent filtration. Sumati et al. prepared the extract of fresh cranberries with methanol. Koo et al. [28], Duarte et al. [32], and Feng et al. [37] determined the composition of PAC by high-performance liquid chromatography (HPLC)—mass spectrometry (MS) with photodiode/electrochemical detection and matrix-assisted laser desorption/ionization (MALDI) time-of-flight (TOF) and was confirmed using LC–MS-MS in product ion scanning mode followed by multiple reaction monitoring (MRM) scanning. Gregoire et al. [39] isolated phenolic acids, flavonols and PACs by semi-preparative HPLC and the isolated compounds were characterized by HPLC–MS. Philip et al. [7], Zhao et al. [33], Furiga et al. [34], Prince et al. [35], and Smullen et al. studies [41] were based on manufacturer-supplied information on polyphenol concentrations in cranberry extracts [7, 35] or GSE [33, 34, 41]. Daglia et al. [31] performed dealcoholization of red wine and fractionation of dealcoholized red wine components such as PACs by Solid Phase Extraction and Gel Filtration Chromatography. Yamanaka et al. [42] dialyzed powdered cranberry juice against distilled water with a molecular mass cut-off point of 14,000. Elgamily et al. [43] full scan chromatograms with subsequent hydrolysis of the samples were used to measure ms ms spectra. Nowaczyk et al. [44] used a constant stirring method with a rotary stirrer followed by centrifugation, decantation and rotary evaporation.

#### Determination of antimicrobial activity

The Minimum Inhibitory Concentration (MIC) was defined by the authors [7, 26, 27, 29, 31, 33, 34, 36, 39, 41, 43, 44] as the minimum concentration of the antimicrobial substance that inhibits the visible growth of the bacteria after incubation. The antibacterial assay was generally conducted using the macrodilution method with agar in petri dishes [26, 30, 34–36, 39, 41, 44], or the microdilution method [39] in liquid medium such as trypticase soy broth [29] or BHI [27, 35, 40] or Mueller–Hinton broth [41] in a 96-well titre plate, and the MIC was evaluated by counting colony-forming units (CFU/mL). The two controls used in the studies to determine the MIC were positive control (MH broth with bacterial suspension) and negative control (MH broth without antimicrobial and without bacterial suspension). The Minimum Bactericidal Concentration was defined as the minimum antimicrobial concentration that eliminated more than 99.9% of viable microorganisms after a given incubation time. Sumati et al. [29] evaluated the ability of cranberry extract to induce cytotoxicity against microorganisms by means of the MTT assay that is based on mitochondrial metabolism. Philip et al. [7] evaluated the ecological effects of extracts on polymicrobial biofilms using real-time quantitative polymerase chain reaction (qPCR) assay to determine the bacterial load, considering 14 bacterial species of interest. Prince et al. [35] performed a checkerboard assay to assess zones of inhibition to determine bacterial growth. Finally, Net et al. [40] determined the inhibition capacity of cranberry fractions in influenza-induced hemagglutination.

#### Effect on biofilm adhesion

The most widely used method to evaluate the effect of test substances on stable biofilm formation was culturing *S. mutans* strains on plastic [24, 27, 31, 40], glass (42), or on hydroxyapatite surfaces (sHA) coated with human saliva obtained from healthy donors [7, 25, 28, 30, 32, 37, 38, 42]. Several studies have used the crystal violet staining method for culture or biofilm staining, with different concentrations and time incubation: 0.1% for 15 min [7, 24, 25, 31], 0.5% for 30 min [27, 29], and 45 min without reporting concentration [40], staining with ethanol [25, 27, 29], acetic acid [7, 40], or ethanol/acetone [31] and the absorbance reading was measured by spectrophotometry. Greene et al. [25] performed the evaluation through optical microscopy and Daglia et al. [31] used a stereomicroscope with radiolabeled strains [31] through a scintillation counter assay, as well as Duarte et al. [32] and Feng et al. [37]. Kokubu et al. [24] used an ATP bioluminescence assay. Zhao et al. [33] induced caries lesion formation in vitro by *S. mutans* biofilm and determined lesion depth and relative optical density using polarized light microscopy and confocal laser scanning microscopy to assess lesion progression. Furiga et al. [34] used a Gram stain to confirm species identity. Philip et al. [38] determined the effects of extracts on *S. mutans* biofilms by evaluating the metabolic activity of biofilm microorganisms with the XTT reduction assay. Yamanaka et al. [42] used direct scintillation counting of the number of attached bacterial cells.

#### Morphological evaluation of biofilms treated with the test substances

To examine the effects of treatments on *S. mutans* biofilms, structural organization, EPS formation, and microbial biovolumes were analyzed by confocal microscopy [7, 34, 37, 38] or by scanning electron microscopy [27, 44].

#### Determination of the effect on microbial glycosyltransferase enzymes (Gtf)

The importance of determining the effect of flavonoids on microbial glycosyltransferase enzymes (Gtf) lies in the fact that they are the enzymes responsible for synthesizing glycans. These glycans are polysaccharides that form the biofilm matrixes. Glycans synthesized by surface-adsorbed GtfB and GtfC provide specific binding sites for bacterial colonization on the tooth surface and with each other. The glycosyltransferase enzymes expression was evaluated by Feng [37] using the RT-qPCR technique and its activity was measured by Duarte et al. [32] and Gregoire et al. [39] by incorporating (14C) glucose into labeled sucrose in the glycans. Koo et al. [28] determined it by means of the enzymes in solution or by adsorbing it on saliva-coated hydroxyapatite beads free of Gtf activity, in the presence of test agents, and Furiga et al. [34] determined it by measuring the amount of reducing sugars released by a dinitrosalicylic acid assay, with fructose as a standard, and by the amount of insoluble glycan synthesized.

#### Effects of the extracts on the drop in glycolytic pH by *S. mutans*

Koo et al. [28] and Duarte et al. [32] evaluated the effects of cranberry PAC on glycolysis by standard pH drop using suspensions of cells exposed to test agents spiked with glucose and assessed for pH drop by electrode pH glass. Philip et al. [7, 38] measured acidogenicity using the lactate dehydrogenase assay to determine the concentrations of lactic acid formed by biofilms.

#### Quantification of the effective antioxidant activity of the compounds

Furiga et al. [34] determined the Trolox equivalent antioxidant capacity (TEAC), which is based on the ability of a sample to scavenge the ABTS radical cation, [2,2′-azinobis (3-ethylbenzothiazoline-6-sulfonic) diammonium salt], compared to the standard antioxidant Trolox [6-hydroxy-2,5,7,8-tetramethyl chroman-2-carboxylic acid]. Net et al. [40] used the luminol-dependent chemiluminescence assay to estimate the oxidant scavenging capacities (OSA) of cranberry fractions in contact with the bacterial surface.

### Test results

#### Effect of extracts on bacterial growth inhibition (MIC, MBC)

Kokubu et al. [24] showed a reduction in the bioactivity of *S. mutans* at concentrations of 0.5. 1.0 and 2.0 mg/mL of cranberry extract. Swadas et al. [26] reported an inhibition of bacterial activity at concentrations of 250 and 500 mg/mL of GSE, while the concentration of 125 mg/mL did not show significant antibacterial activity. Zhao et al. [33] demonstrated bacterial inhibition at a concentration of 4 mg/mL of GSE. According to Ikai et al. [36] 2 mg/mL PACs from cranberry extract significantly increased the bactericidal activity of photoirradiated H_2_O_2_ at concentrations of 80 and 320 nM. Elgamily et al. [43] showed an inhibition of bacterial growth at concentrations of 0.5 and 0.25 mL/mL of cranberry juice and at concentrations of 0.50 mL/mL of functional cranberry drink.

Singhal et al. [27] reported an MIC of 12.5 mg/dL for the ethanolic extract of cranberry, and a MBC of 25 mg/dL. Sumati et al. [29] determined a MIC of 60 mg/mL for cranberry extract, which was characterized as having a bactericidal effect. Abu-obaid et al. [30] showed that the effect of the MIC and MBC of cranberry extract was like that of chlorhexidine and that when these two substances were combined, its effect increased twice and was even superior to that of treatment with chlorhexidine plus alcohol. Daglia et al. [31] reported an MIC of 19.07 mg/mL for GSE dealcoholized wine and 2.53 mg/mL for polymeric PAC eluted with 15 mL of methanol. Furiga et al. [34] for their part, recorded a MIC of 1000 μg/mL of GSE, a concentration at which no bacterial growth was shown after 24 h, and a MBC of 4000 μg/mL, a concentration at which reduction of 99.9% bacterial count was observed. Prince et al. [35] demonstrated by means of the checkerboard test, that the MIC of cranberry extract in subcritical water of presscake with tannase and in resin with tannase was 0.75 mg/mL, while the resin extract was 0.75 mg/mL to 0.5 mg/mL. Smullen et al. [41] determined an MIC for the red GSE extract of 0.5 mg/mL and 1 mg/mL for inhibition of a procyanidin polymer fraction.

#### Effect of extracts on biofilm formation

Kokubu et al. [24] reported a significant decrease in biofilm formation at concentrations of 0.5 and 1.0 mg/mL of cranberry extract in a 1000 mL dilution of 70% ethanol. Greene et al. [25] found that at concentrations of 0.5 and 5 mg/mL of cranberry extract alone, or + C_18_H_36_O_2_ + polyvinylpyrrolidone, or + C_18_H_36_O_2_ + ethyl lauroyl arginate, a reduction in the formation of biofilms. Singhal et al. [27] indicated that after 24 h of treatment, 16.67 mg/dL and 20.83 mg/dL of cranberry extract reduced 50% and 70% of the preformed biofilm, respectively. According to Sumathi et al. [29], 50 mg/mL of cranberry extract acted as a potent antimicrobial agent by inhibiting biofilm formation by 95.2% after 24 h of treatment. Philip et al. [7] demonstrated a significant decrease (51%) in the biofilm formation at 500 μg/mL of cranberry extract. Daglia et al. [31] on the other hand, stated that the polymeric PAC of the GSE extract with 15 mL of methanol had the ability to reduce biofilm formation by 89%, while dealcoholized wine reduced it by 79%. Zhao et al. [33] reported that 4 mg/mL of GSE extract had the ability to inhibit biofilm formation.

Furiga et al. [34] found that 2000 μg/mL GSE + 10.2 mg/mL Fluorinol contributed to decrease the number of microcolonies. According to Ikai et al. [36], there was a significant decrease in colony count when cultures were treated with 8 mg/mL PAC and 500 nM H_2_O_2_. Feng et al. [37] demonstrated a decrease in the biofilm of 4.3 μm^3^ for the treatment group, compared to 28.9 μm^3^ for the control group. Philip et al. [38] evaluated different concentrations, thus, at 500 μg/mL, a 32% biofilm reduction is generated, at 250 μg/mL 29%, while at 125 μg/mL the reduction is minimal (14%). Net et al. [40] found biofilm reduction at concentrations as low as 80 μg/mL with non-dialyzable cranberry plus acetone, while non-dialyzable cranberry only produced a significant decrease at a concentration of 320 μg/mL. Smullen et al. [41] reported that GSE decreases biofilm formation at 30 min of treatment. Yamanaka et al. [42] indicated an inhibition in the formation of biofilms at concentrations of 100 μg/mL and 500 μg/mL at 25% KCl. Finally, Nowaczyk et al. [44] found a 68% decrease in bacterial colony formation at concentrations of 50 μg/2 ml of probiotic candy-GSE with distilled wáter.

#### Antioxidant capacity of the test extracts

Furiga et al. [34] in their study of the equivalent antioxidant capacity of Trolox (TEAC), demonstrate that GSE was the group with the highest antioxidant capacity (6.99 μg/mL), compared to Eludril Daily (6.95 μg/mL), ascorbic acid (5.70 μg/mL) and fluorinol (0.01 μg/mL), which offers a high degree of protection to the organism against the damage of free radicals. Additionally, Neto et al. [40] demonstrated that polyphenols in the studied cranberry fractions of non-dialyzed material, were able to exhibit antioxidant properties. These experiments indicated that the NDMac fraction (eluted in acetone) was about twice as rich in polyphenols and showed twice as higher antioxidant activity in solution or when bound to bacterial surfaces as the NDMet fraction (eluted in ethanol).

#### Effect of the extracts on the expression of GtfB and C. F-ATPase

Koo et al. [28] demonstrated a reduction in the number of extracellular EPSs from 35 to 40%. Additionally, the PACs inhibited the activity of the absorbed GtfB and reduced it to between 40 and 70%. Philip et al. [7] showed a significant decrease in EPS formation with the cranberry extract treatment (1.14 μm^3^/μm^2^) compared to the control (2.64 μm^3^/μm^2^). Duarte et al. [32] reported that treatment with anthocyanidin + flavonol inhibited F-ATPase activity by 85%, and this treatment caused the greatest inhibition of GtfB activity (70%), followed by treatment with PACs + anthocyanidin (50%) and PACs + flavonol (45%). Furiga et al. [34] showed Gtfs inhibition of 43.9% with GSE treatment, with 65.7% inhibition for GSE combined with fluorinol. Feng et al. [37] reported a decrease of 19.8 μm^3^ of EPS, unlike the control group, which showed a decrease of 68.1 μm^3^. On the one hand, Gregoire et al. [39] reported an inhibition of the enzymatic activity of the proton-translocating F-ATPase at 500 μmol from 18 to 33%, with myricetin and procyanidin A2 being the most inhibitory. On the other hand, it showed a 20% to 35% reduction of glycan synthesis by GftB at a concentration of 500 μmol, with procyanidin A2 being the most effective.

#### Morphological changes associated with exposure to flavonoids

Singhal et al. [27] reported that with the increase in cranberry concentration, the integrity of the biofilm is altered, an effect characterized by agglutination and cell degradation at high concentrations. Philip et al. [7] found inhibition of polymicrobial biofilm biomass at 96 h, with a reduction of 38% compared to the control group. Furiga et al. [34] reported that with 2000 μg/mL of GSE there was a decrease in the number of microcolonies and in the thickness of biofilms. Feng et al. [37] reported defective biofilm accumulation and altered architecture, as well as impaired EPS matrix development with cranberry treatment. Philip et al. [38] stated that cranberry treatment resulted in reduced biovolume and less compaction of exposed biofilms. Finally, Nowaczyk et al. [44] report a significantly altered structure of the bacteria cells with a concentration of 50 μg / 2 ml of probiotic candy-GSE.

#### Effects of extracts on pH change

Koo et al. [28] showed that after 4 h of incubation with PAC treatment of cranberry extract, the pH remained slightly higher (5.6) than that of the group containing no extract (5.2). Philip et al. [7] found a significant difference in the production of lactic acid in the control group without extract, which was 11.2 mM/L between the experimental group treated with cranberry extract, in which it was 6.2 mM/L, showing a 44% reduction. Duarte et al. [32] reported a final pH of 4.7 to 4.9 with the PAC of cranberry extract, which was different from the control group without extract, and was 3.7. Philip et al. [38] showed that at a concentration of 500 μg/mL of cranberry extract, there was a 40% reduction in lactic acid production by *S. mutans*. Finally, Gregoire et al. [39] stated that cranberry extract flavonoids, such as myricetin and procyanidin A2, significantly disrupted the glycolytic pH drop by *S. mutans.*

#### Effect of extracts on bacterial adhesion

Greene et al. [25] evaluated the adhesion of microencapsulated mouthwashes to hydroxyapatite (HA), formulations with cranberry extract were able to adhere to the HA disks after 24 h of incubation and prevent the biofilm formation. Daglia et al. [31] demonstrated inhibition of *S. mutans* adhesion to hydroxyapatite of 81.5% with GSE at a concentration of 0.125 mg/mL with an elution of polymeric proanthocyanidin with 15 mL of methanol (SPE F4), while dealcoholized wine inhibited 25% adhesion at a concentration of 0.25 mg/mL. Feng et al. [37] for their part, reported an inhibition of bacterial adhesion due to the reduction in EPS synthesis, associated with the inhibition of the enzymatic activity of GtfB and GtfC generated by the PAC extract. Finally, Smullen et al. [41] reported that at an MIC of 0.5 mg/mL, the adhesion capacity of *S. mutans* is decreased. Yamanaka et al. [42] showed a significant reduction of initial colonization by *S. mutans* of 40–60%, the inhibitory activity was related to the reduction of the hydrophobicity.

#### Induction of cell death and cytotoxicity

Singhal et al. [27] found a reduction in the bacterial count that was dependent on time and extract concentration. After 10 h, the reduction was 50%. Furiga et al. [34] showed a very low level of cell damage (< 1%), with few visible dead cells.

## Discussion

### Summary of the main results

The studies included in this review demonstrated an inhibitory effect on the bacterial growth of *S. mutans*, of cranberry extract in ranges from 0.5 mg/mL to 25 mg/mL, and of GSE from 0.5 mg/mL to 250 mg/mL. Additionally, a reduction capacity of the extracts or their fractions in the formation of biofilms, decrease in the biomass of the polymicrobial biofilm, deregulation of the expression of GtfB and C, and buffering of the drop in pH were observed, in addition to an adequate antioxidant activity associated with the content of polyphenols. These positive effects have been related to the decrease in virulence factors of the oral pathogen, and the inhibition of the formation of insoluble polysaccharides in the extracellular matrix, which prevents glycan-mediated adhesion, cohesion, and aggregation of *S. mutans* [28]. This may suggest that these natural extracts could play an important role in the prevention of cariogenic bacterial colonization, as well as induce a decrease in their microbiological activity.

### Quality of the evidence, limitations, and potential biases in the review

The results of the studies included in this review correspond to those of in vitro studies, which must be interpreted with caution, since they cannot fully reflect all the circumstances of a clinical situation. Additionally, the differences in the methodologies used and variations in the characteristics of the laboratory tests (type of extracts, methods of obtaining, separation or not of fractions, concentrations, etc.) can yield heterogeneous results, so only one test was performed according to individual analysis of studies. According to the bias assessment, all the articles (100%) had a medium and low risk of bias (Table 3), which allowed for a compilation of reliable results that may be useful to guide in vivo studies and improve research methodologies for the analysis of new alternatives for microbiological control in the formation of dental biofilms. However, it is important to recognize that while the studied compounds may have demonstrated effects on *S. mutans*, dental caries is a polymicrobial disease. In addition to being biofilm-mediated, it is also a diet-modulated pathology that is multifactorial, non-communicable, and dynamic. Therefore, it is necessary to consider the analysis of other additional factors for the control of dental caries, as it is determined by biological, behavioral, psychosocial, and environmental factors [1]. This recognition underscores the complexity of dental caries etiology and emphasizes the need for comprehensive approaches that address multiple contributing factors beyond the singular focus on specific bacterial strains.

### Agreements and disagreements with other studies or reviews

Biofilms are the natural habitat of *S. mutans*, with their maturation, the bacteria are provided with greater anchorage and protection, and play a biologically active role that allows them to retain nutrients and water for their metabolism [46]. According to the analyzed studies, the above-mentioned biofilm is affected by an inhibition of 34% to 51% in considerable concentrations of PAC, as well as a reduction in the structural organization of already formed biofilms. In addition, morphological alterations such as a decrease in the thickness of the biofilm, defective architecture, agglutination, and interruption in integrity were reported, which consequently result in a disorganized biofilm that is susceptible to degradation and with little response capacity.

One of the factors that the authors have given high relevance to in the analysis of the effectiveness of the extracts has been their effect on changes in pH since these have managed to create an adequate cellular environment to inhibit bacterial proliferation. The flavonoids contained in the extracts such as PACs increase the pH from 4.7 to 5.6. This generates a propitious effect for the inhibition of bacterial growth since it has been previously reported that environments with a pH lower than 5.0 are suitable for bacterial proliferation, and that the pathogen can even keep its cytoplasm alkaline at lower pH (2.5 to 3.0) [47]. Additionally, it has been reported that raising the pH value will result in the bacteria not having the ability to induce repeated changes in the fatty acids of their membrane [48].

To make the environment conducive to bacterial proliferation, the action of F-ATPase is essential. This enzyme plays an important role in the production of ATP in *S. mutans*, for the protection and survival of the pathogen in conditions of sudden change to the acidic environment, inhibition of ATP reduces its resistance and, once again, conditions cause decrease in its proliferation [3]. One study reported the inhibition of this proton-translocating enzyme between two complexes, the hydrophobic proton-conducting F0 and the F1 consisting of the water-soluble catalytic site, with PAC treatment, by binding to the F1 complex [32]. The inhibition effect by exposure to PAC in the different studies ranges between 18 and 85%.

Another important factor is the effect on Gtfs and bacterial adhesion. A decrease in the expression of GtfB between 40 and 70% was reported, which produced an impact on the reduction of 35 to 40% in the number of EPS generated for biofilm formation. This effect has been associated with a decrease in bacterial colonization capacity, since the GtfB enzyme, capable of synthesizing glucans that are insoluble in water, has the vital function of interacting with other bacteria of the same species, which produces environments rich in glucan that allow the adhesion of more pathogens [49]. The studies reported that the decrease in the expression of GtfC leads to a decrease in the primary colonization of S*. mutans*, since this enzyme synthesizes soluble and insoluble glucans that are rich in glucose, presenting the highest affinity for hydroxyapatite crystals of the three types of Gtfs [50]. Likewise, this adhesion to the hydroxyapatite crystals was altered in in vitro studies by an inhibition of 81.5% by GSE, and by a decrease in the direct production of glucans.

The relationship between the structure of the compound and its antimicrobial activity has been investigated. Core structures with 3,4,5-trihydroxyphenyl groups found in epigallocatechin, epigallocatechin-3-ogallate, castalagin, and prodelphinidin may be important for antibacterial activity. It indicated that the number of hydroxyls and the degree of polymerization could be fundamental for the antimicrobial activity of phenolic compounds. Regarding the antimicrobial activity of flavonoids, the importance of the epicatechin subunit and the presence of orthohydroxyl groups in the B ring for radical scavenging and modulation of the immune response have been reported. Different concentrations of anthocyanins and flavonols markedly decreased the activity of Gtfs B and C in *S. mutans* cultures [51]. The results suggest that the conjugation of phenolic compounds and proteins in microorganisms, especially key enzymes for their proliferation and adhesion, could be an important way to inhibit the growth of microorganisms.

### Implications for practice

Although this is a systematic review of in vitro studies, it is expected that the compilation of scientific evidence will contribute to developing novel alternatives such as antimicrobials of natural origin, by incorporating agents rich in polyphenols extracted from fruits such as cranberries and the grape seeds. This can help to reduce the virulence factors of *S. mutans*, to generate a favorable environment that inhibits its primary colonization capacity and subsequent formation of cariogenic multispecies biofilms. These alternatives, in addition to demonstrating their high effectiveness, have few side effects and promote the use of natural resources for human health.

### Implications for future research

Despite the number of in vitro studies included in this review, the need for more such studies to evaluate the antimicrobial effects of flavonoids, is evident. Corroborate the results already exposed to carry out the transfer of its use to the clinic. Additionally, the effects of the individual active compounds or fractions of the extracts could be further explored. Finally, it is necessary to develop more efficient extraction processes for natural compounds and to insist on incorporating them into daily oral hygiene methods.

## Conclusions

The overall results demonstrated that cranberry and grape seed extracts or their respective fractions were effective in altering the virulence properties of *S. mutans*. The data showed that PACs are the components of cranberry and grape seeds that are active against *S. mutans*. The main routes through which these substances affect the virulence of *S. mutans* may be the inhibition of the synthesis of insoluble glucans by GTF B and C adsorbed on the surface, inhibition of proton-translocating F-ATPase activity, and disruption of acidogenesis. This suggests the antimicrobial potential of the polyphenols contained in these fruits, as agents capable of modulating the pathogenicity of cariogenic biofilms.

### Supplementary Information

**Supplementary Material 1.**

## Data Availability

All data generated or analysed during this study are included in this published article.

## Abbreviations

ATCC: American Type Culture Collection
EPS: Extracellular Polysaccharides
GSE: Grape Seed Extract
Gtf: Glycosyltransferase
HPLC: High-Performance Liquid Chromatography
IPS: Intracellular Polysaccharides
MBC: Minimum Bactericidal Concentration
MIC: Minimum Inhibitory Concentration
MS: Mass Spectrometry
PACs: Proanthocyanidins
sHA: Hydroxyapatite surfaces
SPE: Solid Phase Extraction
TEAC: Trolox Equivalent Antioxidant Capacit
