# Supplementary material for: Effect of flavonoids from grape seed and cranberry extracts on the microbiological activity of Streptococcus mutans: a systematic review of in vitro studies
Source: BMC Oral Health. 2024 Jun 5;24:662. doi: 10.1186/s12903-024-04263-0 (PMC11155149; doi:10.1186/s12903-024-04263-0)
Supplement: Supplementary file 1 — Supplementary Material 1. [file 12903_2024_4263_MOESM1_ESM.docx]

| Search # 1  **Additional file 1.** Search algorithms in the different databases. | |
| --- | --- |
| Database | MEDLINE (Vía PubMed) |
| Last search update | 30/12/2023 |
| Time limit | None |
| Language limits | None |
| Other limits | None |
| Search strategy | \| # \| Query \| Results \| \| --- \| --- \| --- \| \| 1 \| Streptococcus mutans[MeSH Terms] \| 10,184 \| \| 2 \| Streptococcus mutans \| 14,009 \| \| 3 \| Biofilms[MeSH Terms] \| 44,684 \| \| 4 \| Biofilm* \| 77,793 \| \| 5 \| (((Streptococcus mutans[MeSH Terms]) OR (Streptococcus mutans)) OR (Biofilms[MeSH Terms])) OR (Biofilm*) \| 89,151 \| \| 6 \| microbiological activity \| 334,543 \| \| 7 \| Minimum Bactericidal Concentration \| 5,209 \| \| 8 \| microbial sensitivity tests[MeSH Terms] \| 153,357 \| \| 9 \| Minimum Inhibitory Concentration \| 165,319 \| \| 10 \| Microbial Sensitivity Tests \| 155,020 \| \| 11 \| Concentration, Minimum Inhibitory \| 165,319 \| \| 12 \| Dental caries[MeSH Terms] \| 50,651 \| \| 13 \| dental caries \| 66,792 \| \| 14 \| Dental Decay \| 68,022 \| \| 15 \| ((((((((microbiological activity) OR (Minimum Bactericidal Concentration)) OR (microbial sensitivity tests[MeSH Terms])) OR (Minimum Inhibitory Concentration)) OR (Microbial Sensitivity Tests)) OR (Concentration, Minimum Inhibitory)) OR (Dental caries[MeSH Terms])) OR (dental caries)) OR (Dental Decay) \| 538,489 \| \| 16 \| Grape Seed Extract[MeSH Terms] \| 969 \| \| 17 \| Grape Seed Extract \| 1,750 \| \| 18 \| Grape Seed* \| 4,037 \| \| 19 \| cranberry extract* \| 874 \| \| 20 \| lingonberry extract* \| 140 \| \| 21 \| lingonberry \| 363 \| \| 22 \| cranberry \| 2,260 \| \| 23 \| ((((((Grape Seed Extract[MeSH Terms]) OR (Grape Seed Extract)) OR (Grape Seed*)) OR (cranberry extract*)) OR (lingonberry extract*)) OR (lingonberry)) OR (cranberry) \| 6,522 \| \| 24 \| ((((Streptococcus mutans[MeSH Terms]) OR (Streptococcus mutans)) OR (Biofilms[MeSH Terms])) OR (Biofilm*)) AND (((((((Grape Seed Extract[MeSH Terms]) OR (Grape Seed Extract)) OR (Grape Seed*)) OR (cranberry extract*)) OR (lingonberry extract*)) OR (lingonberry)) OR (cranberry)) \| 149 \| \| 25 \| (((((Streptococcus mutans[MeSH Terms]) OR (Streptococcus mutans)) OR (Biofilms[MeSH Terms])) OR (Biofilm*)) AND (((((((Grape Seed Extract[MeSH Terms]) OR (Grape Seed Extract)) OR (Grape Seed*)) OR (cranberry extract*)) OR (lingonberry extract*)) OR (lingonberry)) OR (cranberry))) AND (((((((((microbiological activity) OR (Minimum Bactericidal Concentration)) OR (microbial sensitivity tests[MeSH Terms])) OR (Minimum Inhibitory Concentration)) OR (Microbial Sensitivity Tests)) OR (Concentration, Minimum Inhibitory)) OR (Dental caries[MeSH Terms])) OR (dental caries)) OR (Dental Decay)) \| 82 \| |
| **Identified documents** | **82** |

| Search # 2 | |
| --- | --- |
| Database | EMBASE (Vía Elsevier) |
| Last search update | 30/12/2023 |
| Time limit | None |
| Language limits | None |
| Other limits | None |
| Search strategy | \| # \| Query \| Results \| \| --- \| --- \| --- \| \| 1 \| 'streptococcus mutans'/exp OR 'streptococcus mutans' \| 16,411 \| \| 2 \| 'biofilms'/exp \| 74,048 \| \| 3 \| 'biofilm' \| 91,550 \| \| 4 \| #1 OR #2 OR #3 \| 104,190 \| \| 5 \| 'microbiological activity' \| 600 \| \| 6 \| 'minimum bactericidal concentration' \| 8,963 \| \| 7 \| 'microbial sensitivity tests'/exp \| 14,743 \| \| 8 \| 'microbial sensitivity test' \| 14,777 \| \| 9 \| 'minimum inhibitory concentration' \| 140,177 \| \| 10 \| 'concentration, minimum inhibitory' \| 46 \| \| 11 \| 'dental caries'/exp \| 64,100 \| \| 12 \| 'dental caries' \| 68,375 \| \| 13 \| 'dental decay' \| 978 \| \| 14 \| #5 OR #6 OR #7 OR #8 OR #9 OR #10 OR #11 OR #12 OR #13 \| 222,692 \| \| 15 \| 'grape seed extract'/exp \| 2,121 \| \| 16 \| 'grape seed extract' \| 2,362 \| \| 17 \| 'grape seed' \| 3,449 \| \| 18 \| 'cranberry extract' \| 766 \| \| 19 \| 'cranberry extract*' \| 802 \| \| 20 \| 'lingonberry extract' \| 37 \| \| 21 \| 'lingonberry' \| 385 \| \| 22 \| 'cranberry' \| 3,534 \| \| 23 \| #15 OR #16 OR #17 OR #18 OR #19 OR #20 OR #21 OR #22 \| 7,203 \| \| 24 \| #4 AND #23 \| 203 \| \| 25 \| #14 AND #24 \| 76 \| |
| Identified documents | **76** |

| Search # 3 | |
| --- | --- |
| Database | SCOPUS (Vía Scopus) |
| Last search update | 30/12/2023 |
| Time limit | None |
| Language limits | None |
| Other limits | None |
| Search strategy | \| # \| Query \| Results \| \| --- \| --- \| --- \| \| 1 \| TITLE-ABS-KEY ( ( "streptococcus mutans" OR "s. mutans" OR "biofilm" OR "biofilms" OR "dental plaque" OR "dental biofilms" OR "dental caries" ) ) \| 217,183 \| \| 2 \| TITLE-ABS-KEY ( ( "cranberry" OR "lingonberry" OR "lingonberry extract*" OR "cranberry extract*" OR "grape seed*" OR "grape seed extract" ) ) \| 10,743 \| \| 3 \| TITLE-ABS-KEY ( "microbiological activity" OR "minimum bactericidal concentration" OR "microbial sensitivity tests" OR "minimum inhibitory concentration" OR "microbial sensitivity tests" OR "concentration minimum inhibitory" ) \| 229,570 \| \| 4 \| 1 AND 2 \| 276 \| \| 5 \| 4 AND 3 \| 48 \| \| Final \| ( ( TITLE-ABS-KEY ( ( "streptococcus mutans" OR "s. mutans" OR "biofilm" OR "biofilms" OR "dental plaque" OR "dental biofilms" OR "dental caries" ) ) ) AND ( TITLE-ABS-KEY ( ( "cranberry" OR "lingonberry" OR "lingonberry extract*" OR "cranberry extract*" OR "grape seed*" OR "grape seed extract" ) ) ) ) AND ( TITLE-ABS-KEY ( "microbiological activity" OR "minimum bactericidal concentration" OR "microbial sensitivity tests" OR "minimum inhibitory concentration" OR "microbial sensitivity tests" OR "concentration minimum inhibitory" ) ) \| 48 \| |
| Identified documents | **48** |

| Supplementary Searches (30/12/2023) | | | |
| --- | --- | --- | --- |
| Databases | Search algorithm | Limitation | Results |
| Science Direct | ("streptococcus mutans" OR Biofilm OR "dental caries") AND (proanthocyanidins OR "grape seed extract" OR "lingonberry extract" OR "cranberry extract") | Research Article  Thematic area: medicine and dentistry. Immunology and Microbiology | 83 |
| Web of Science | (AB= (("streptococcus mutans" OR Biofilm OR "dental caries") AND (proanthocyanidins OR "grape seed extract" OR "lingonberry extract" OR "cranberry extract"))) | Advanced search  Language: English | 75 |
| EBSCO | ("streptococcus mutans" OR Biofilm OR "dental caries") AND (proanthocyanidins OR "grape seed extract" OR "lingonberry extract" OR "cranberry extract") | Source: Dentistry and Oral Sciences | 28 |
| Cochrane | ("streptococcus mutans" OR Biofilm OR "dental caries") AND (proanthocyanidins OR "grape seed extract" OR "lingonberry extract" OR "cranberry extract") |  | 28 |
| Identified documents | **214** | | |
